# Autoantibody-Specific Signalling in Pemphigus

**DOI:** 10.3389/fmed.2021.701809

**Published:** 2021-08-09

**Authors:** Thomas Schmitt, Jens Waschke

**Affiliations:** Ludwig-Maximilian-Universität München, Anatomische Anstalt, Lehrstuhl Anatomie I – Vegetative Anatomie, Munich, Germany

**Keywords:** adhesion, pemphigus, signalling, autoantibodies, autoimmune blistering disease

## Abstract

Pemphigus is a severe autoimmune disease impairing barrier functions of epidermis and mucosa. Autoantibodies primarily target the desmosomal adhesion molecules desmoglein (Dsg) 1 and Dsg 3 and induce loss of desmosomal adhesion. Strikingly, autoantibody profiles in pemphigus correlate with clinical phenotypes. Mucosal-dominant pemphigus vulgaris (PV) is characterised by autoantibodies (PV-IgG) against Dsg3 whereas epidermal blistering in PV and pemphigus foliaceus (PF) is associated with autoantibodies against Dsg1. Therapy in pemphigus is evolving towards specific suppression of autoantibody formation and autoantibody depletion. Nevertheless, during the acute phase and relapses of the disease additional treatment options to stabilise desmosomes and thereby rescue keratinocyte adhesion would be beneficial. Therefore, the mechanisms by which autoantibodies interfere with adhesion of desmosomes need to be characterised in detail. Besides direct inhibition of Dsg adhesion, autoantibodies engage signalling pathways interfering with different steps of desmosome turn-over. With this respect, recent data indicate that autoantibodies induce separate signalling responses in keratinocytes *via* specific signalling complexes organised by Dsg1 and Dsg3 which transfer the signal of autoantibody binding into the cell. This hypothesis may also explain the different clinical pemphigus phenotypes.

## Introduction

Pemphigus is a severe autoimmune blistering skin disease disrupting desmosomes and thereby affecting the epidermis of the skin and the mucosa (1, 2). Desmosomes are important epithelial cell-cell contacts providing high mechanical stability (3–5). Disruption of desmosomes causes the cells to detach from each other, leading to acantholysis. The desmosomal transmembrane cadherins comprise desmogleins (Dsg) 1–4 and desmocollins (Dsc) 1–3 (3). They are connected to the plaque proteins plakoglobin (PG), plakophilin (PKP) 1–3, and desmoplakin (DP). The plaque anchors the desmosomes to the keratin filament cytoskeleton (6–11). The composition of desmosomes varies within tissues and different layers of the epidermis which is relevant since Dsg isoforms differ in their function to regulate signalling pathways in pemphigus. Dsg2, which is ubiquitously expressed throughout other epithelia, can only be found in the basal layer of neonatal epidermis but is absent in adult epidermis outside of hair follicles (12–14). In contrast, Dsg1 and Dsc1 expression increase whereas the amount of Dsg3 and Dsc3 decrease towards superficial epidermal layers with Dsg3 being absent in the stratum granulosum. Dsg4 on the other hand is missing from basal cells, described to be only found in the granular layer and hair follicles (13). Starting from the granular layer, corneodesmosine starts additionally linking cadherins together in the extracellular space (13). This shift in composition marks the stepwise maturation of the adaptable desmosomes towards stable corneodesmosomes found in the cornified layer. In mucosal epithelium, the situation is slightly different. Dsg2 is found in the basal layers only (15). Dsg1 is less predominant and particularly absent in the basal layer of mucosa whereas Dsg3 is present in all layers (15). Dsg4 can also be found in all layers above the basal layer. In contrast, Dsc1 is not present in oral mucosa while Dsc2 and 3 are present in all layers (3). The expression patterns of desmosomal cadherins in the mucosa are thus more homogeneous compared to epidermis. In neonatal mouse skin, it was shown, that the more ubiquitous distribution of Dsg3 without a distinct enrichtment of Dsg1 in the upper epidermal layers (16).

All pemphigus variants are rather rare and here we focus on the two main variants of the disease pemphigus vulgaris (PV) and pemphigus foliaceus (PF). Incidence and prevalence of PV vary profoundly in different geographical regions as well as ethnicities. In Germany, the prevalence for PV in 2014 was 94.8 per one million population (pmp) (17). It was shown that some genetic variations particularly of the HLA class II (18–24) but also non-HLA genes (23, 25–37), including *DSG3* (38) play a role. Especially, different expression of the transcription factor ST18, which alters signalling pathways including ERK and is associated with apoptosis, was found tocorrelate with increased prevalence in of PV (22, 32, 39, 40). Of course, environmental risk factors might be of importance as well.

For PF, the prevalence was 10.01 pmp (17), with an estimated incidence rate of <1 pmp in the USA and Europe. PF can manifest sporadically, usually at a middle age. It was shown, that genetic-HLA (41–45) and non-HLA markers (28, 46) are relevant as well. However, there are also more frequent endemic PF variants, e.g., in some subtropical areas of Brazil with incidence rates as high as 3–5% and an onset often at a relatively young age (47–52). Other regions such as Tunisia (53) or Colombia (54) show similar variants.

**Figure 1:**
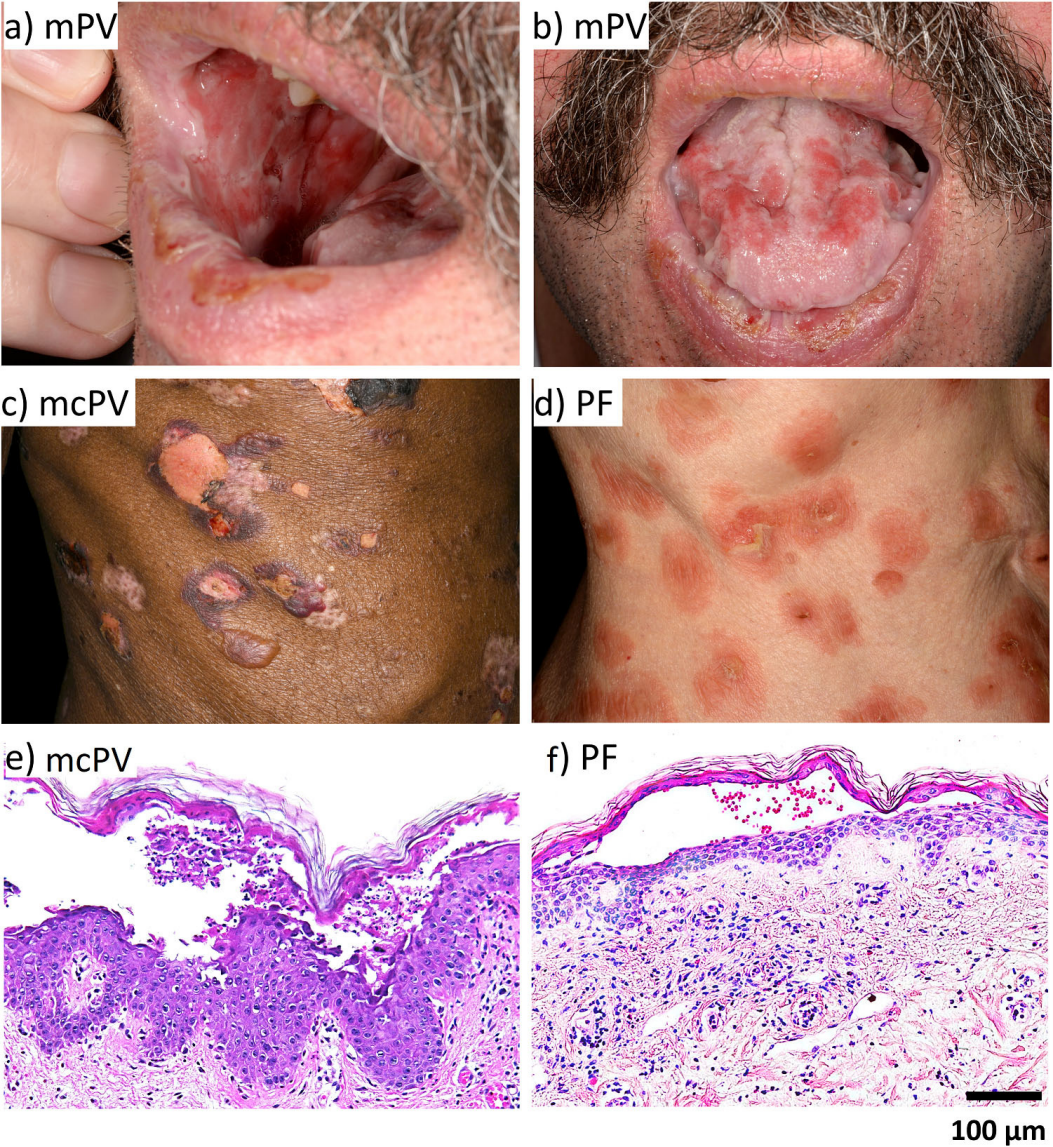
The clinical phenotype and histology of PV and PF. **(a)** mPV. **(b)** mPV. **(c)** mcPV. **(d)** PF. **(e)** mcPV. **(f)** PF.

In most cases, PV initially shows mucosal erosions sometimes spreading to the oesophagus, the airways, the anogenital mucosa (2, 55) and in rare cases even the conjunctiva (56) (Figure 1). In about half of the cases after mucosal erosions additional epidermal lesions develop (57). In contrast, PF affects the more superficial layers of the epidermis only and is less severe (2, 58, 59), however a spread to deeper layers as well as the mucosa and thus progression to PV is possible (60).

For diagnosis of pemphigus, the specific histology with separation at the suprabasal layer for PV and at the granular layer for PF is important (3, 61) (Figure 1). The second specific diagnostic marker are the circulating auto-antibodies against Dsg1 and Dsg3 and in some cases against Dsc3 (57, 61–66), Dsc1 (65), or Dsc2 (66, 67) as well as the accumulation of auto-antibodies in perilesional skin (55, 57). Auto-antibody titers often correlate with disease severity (57, 61, 68, 69).

Pemphigus is a very severe disease with a mortality of around 71% if untreated. Secondary infections due to the disruption of the skin barrier are the most severe problem (58, 70). All treatment strategies aim to reduce the levels of circulating pathogenic autoantibodies. With the introduction of systemic corticosteroid therapy for unspecific immunosuppression, the mortality rate was significantly improved to about 21% (71). However, especially under long-time therapy, these medications cause many side effects including increased infection tendency, osteoporosis, low blood sugar, weight gain arterial hypertonia, cataracts, and glaucoma. These side effects can be reduced or delayed by combination therapy with other immune-suppressive agents or antibodies such as Rituximab, which depletes pathogenic B cells, to reduce the steroid dose (72–75). Current combination therapies reduce the mortality to about 5–10% with death mostly resulting from adverse drug side effects (76). These agents usually show less severe but nonetheless considerable side effects including liver toxicity, anaemia, neutropenia, increased infection risk, cold-like symptoms, increased thirst and urination or in some rare worst cases neurological symptoms reaching from sight loss to impaired movement or speech. Patients might also develop an insensitivity to many of these agents (73, 77).

Novel therapy options are for example intravenous injection of high doses of IgG of healthy donors (78, 79), competitively blocking Fc-receptors of inflammatory cells, reducing B-cell response and inducing autoantibody catabolism (80). Another successful concept is plasmapheresis, where the patient's pathogenic autoantibodies are removed (76). In recent years, plasmapheresis is replaced by immunoapheresis, depleting the patient's own autoantibodies by protein A immunoadsorbtion and thus causing less side-effects (81, 82). However, all these therapies have their own drawbacks, including the high costs (83), availability of donor IgG (84) and the need for regular long treatment sessions. Currently the treatment with corticosteroids in combination with immune suppressive agents and Rituximab is still the standard method (72, 85, 86). Furthermore, it is known that not all patients respond to these therapy concepts.

There are some experimental therapy approaches including humanised anti-CD20 antibodies other than Rituximab, designed to be more specific, more potent and/or less immunogenic (87–89). Antibodies directed against targets other than CD20 are also in development (90–92). A similar approach is targeting B-cell signalling and crosstalk with specific inhibitors (93, 94). Furthermore, IgE signalling *via* IL-4 on Th2 cells which are involved in pemphigus pathology are a novel target (95–97). Targeting Abl-thyrosine kinase-mediated extravasation of autoantibodies into the skin seems to be a promising approach, too (98–100). Another target impacting the autoantibody function are Fc receptors, which prevent IgG degradation and increase autoantibody halflife (101–103). Another approach is to restore Dsg tolerance (104, 105) by transferring Foxp3-expressing regulatory T cells which were shown to be markedly reduced in pemphigus (95). A different emerging option is the therapy with chimeric antigen receptor T-cells (CAR-T)/chimeric autoantibody T-cells (CAA-T) which target a specific antigen and kill the target cell. They could thus deplete autoreactive B-cells (106–108).

For all therapy approaches to reduce autoantibody formation one limitation is that time is required until patients benefit. Therefore, for the acute phase of disease as well as for patients suffering from relapses additional therapeutic options directly stabilising keratinocyte desmosomal adhesion would fulfil an unmet medical need. Directly interfering with keratinocyte signalling to stabilise desmosomes and to induce desmosome assembly could thus be a very promising therapeutic target.

## The Role of Autoantibodies

Pathogenic autoantibodies in pemphigus are mostly directed against Dsg1 and Dsg3. Antibodies against other desmosomal and non-desmosomal proteins are also found. However, whilst it was shown that anti-Dsg1 and anti-Dsg3 antibodies are pathogenic, the role of most other antibodies for the pathology of pemphigus is not clear (2, 58, 105–112). One rare exception are the autoantibodie against Dsc isoforms such as Dsc3 which induce a pathology similar to anti-Dsg antibodies (57, 61–67). Nevertheless, it is likely that some of these auto-antibodies may cause additive effects which affect the clinical phenotype (109). To address this possibility, the so-called “multiple hit hypothesis” postulates that for the actual disease to occur the cumulative effects of several of these antibodies against desmosomal and non-desmosomal antigens are needed (110, 111). However, AK23, which is a Dsg3-specific antibody from an active mouse model, and monoclonal autoantibodies targeting Dsg1 isolated from PF patients have been shown to induce pemphigus-typical acantholysis (112, 113). This demonstrates that autoantibodies against Dsg molecules are sufficient to cause pemphigus, at least under experimental conditions.

Pathogenic pemphigus autoantibodies targeting Dsg1 and Dsg3 are mostly directed against EC1 and 2, while antibodies directed for example against EC5 are reported to be non-pathogenic (23, 114). It is theorised that pathogenic pemphigus autoantibodies can evolve from the non-pathogenic antibodies *via* epitope spreading. It is known that non-pathogenic Dsg1 autoantibodies are increased in unaffected individuals of high incidence endemic populations (23, 45, 115, 116). In endemic PF, the subtype of IgG usually was found to be IgG4 whereas antibodies in unaffected individuals were almost exclusively IgG2 (117). However, non-pathogenic and pathogenic antibodies often share the same light and heavy chains (114), providing credence to this theory (23, 95, 114). Epitope spreading from Dsg3 to Dsg1 is described in the relatively frequent transformation from mucosal-dominant (anti-Dsg3-IgG only) to mucocutaneous PV (anti-Dsg1- and anti-Dsg3-IgG). The same is true for a less frequent transformation from PF (anti-Dsg1-IgG only) to PV (anti-Dsg1- and anti-Dsg3-IgG) (58, 70). Similarly, autoantibodies against plaque proteins or Dsc isoforms also support this hyposthesis (95). A further piece of evidence is the observation that the introduction of a single Dsg3-reactive T-cell was sufficient to induce the expression of polyclonal anti-Dsg3 IgG in mice *in vivo* (118).

The importance of these most prevalent pemphigus autoantigens is demonstrated further by models to deplete the expression of the corresponding proteins. It was shown that knockout of Dsg3 in mice alone is sufficient to cause a pemphigus-like phenotype (119–121). Similar results were observed for deletion of Dsc3 (122), the autoantigen of atypical pemphigus (123). Similar results were observed in an active Dsc3 mouse model producing autoantibodies against Dsc3 *in vivo* (124). In contrast, Dsg1 K.O. mice show a complete abrasion of the superficial epidermis comparable to the histology in PF during normal birth and are not viable (125).

## Pathogenesis Induced by Autoantibodies Targeting Dsg1 and Dsg3 as Revealed by Experimental Models

Under experimental conditions, autoantibodies against Dsg1 and Dsg3 have been demonstrated to disturb the amount and localisation of the respective desmosomal cadherins as well as to induce reorganisation of the cytoskeleton. anti-Dsg3-IgGs cause reorganisation and depletion of Dsg3 from the cell surface (126, 127). In the skin of PV patients less Dsg3 compared to healthy individuals was detectable (128). Both PV-IgG (126, 129) and the monoclonal antibody AK23 reduced membranous Dsg3 *in vitro* (130) in human skin *ex vivo* (127) and in neonatal mice *in vivo* (127). Starting at a reduction by about 50%, cell adhesion *in vitro* was significantly reduced (130). For mice injected with PF-IgG a reduction of Dsg1 by about 30% was enough to cause blistering (128). In line with this, blister formation in neonatal mice was inhibited under conditions which blocked the depletion of Dsg3 (127). The internalisation of Dsg3 occurs *via* endocytosis in a complex with PG. Following the internalisation, the proteins are degraded in the lysosome (126).

Despite the well-established importance of Dsg3 depletion in pemphigus pathogenesis it is yet unclear if this is an early or late mechanism (3). Furthermore, it was shown that different Dsg3 fractions exist which behave differently in terms of depletion (129–132). This is important since localisation of desmosomal cadherins is not limited to the desmosomes (133, 134). Rather, extradesmosomal Dsg molecules (135–137) linked to the actin cytoskeleton (138) are reported as well. It was observed that the extradesmosomal Dsg3 is depleted relatively rapidly following exposure to pemphigus autoantibodies whereas it takes much longer until the Dsg3 integrated in desmosomes is notably impacted by pathogenic IgG (129–131, 139). Since it was observed that acantholysis is actually initiated in the spaces between the desmosomes (140, 141) while keratinocytes stay connected punctually *via* the desmosomes (142) which are disrupted later (143–145), it is hypothesised that the exradesmosomal Dsg3 complexes represent desmosome precursors, which are later integrated into desmosomes (135, 146). This process could possibly be disrupted by pathogenic autoantibodies leading to endocytosis and degradation of these precursors (129, 135, 139). Since desmosomes are dependent on a dynamic equilibrium and are constantly remodelled under high molecular fluctuation (147, 148) this would prevent desmosome formation and cause disassembly of existing desmosomes (135, 149). A different hypothesis proposes a membrane-receptor-like function for non-desmosomal Dsg3 (59, 144, 150) suggesting that extradesmosomal and desmosomal cadherins may have distinct roles.

Together with the disorganisation of the desmosome a retraction of keratin filaments from the cell surface occurs (151–153). During this process, keratin filaments detach from the desmosomes (142, 143, 145, 154) and concentrate around the nucleus (141, 153–156). It was shown that the retraction of keratin filaments starts even before the internalisation of Dsg3 and correlates with the time the cell dissociation starts. Keratin filaments show a clear association with Dsg3 reorganisation at the membrane, preceding endocytosis (153) and turnover after PV-IgG treatment (151). This mechanism is thus proposed to play an important role in pemphigus pathogenesis and especially the time of onset before desmosome disassembly suggest keratin filament retraction to be a primary pathogenic mechanism (153). The actin cytoskeleton was also shown to be affected by PV-IgG which may interfere with desmosome assembly (129, 157).

## Mechanisms Causing Acantholysis in Pemphigus

Meanwhile it is widely accepted that the mechanisms underlying loss of cell adhesion in pemphigus comprise both direct inhibition of Dsg interaction by bound autoantibodies and signalling mechanisms regulating keratinocyte adhesion triggered by autoantibody binding (158). In the pioneering studies on pathologic mechanisms direct inhibition of Dsg3 adhesion by steric hindrance was very suggestive to be causal for acantholysis (159) whereas signalling such as PLC-induced Ca^2+^ release (156, 160) was regarded more as secondary bystander effect.

Meanwhile, there are some studies available on steric hindrance of Dsg1 and Dsg3 adhesion, which for technical reasons are mostly carried out under cell-free conditions *in vitro* using bead assays or single-molecule atomic force microscopy (161) but rarely were performed in cultured keratinocytes (Table 1). However, studies using *in vivo* models or human organ culture are lacking which makes it hard to decide whether effects of autoantibodies on recombinant extracellular domains of Dsg1 and Dsg3 can be transferred to the situation in intact skin where desmosomes are formed in a complex multi-step process. In contrast, there is ample evidence for the role of signalling pathways in the regulation of desmosomal adhesion in PV from studies not only performed in cultured cells but also using *in vivo* mouse models and *ex-vivo* human epidermis whereas data for PF are still limited (Table 2).

**Table 1:** Studies on direct inhibition of Dsg1 and Dsg3 interaction in pemphigus.

**PV**	***In vitro* (cell-free)**	***In vitro* (cultured cells)**	***In vivo* (mice)**	***Ex vivo (human)***
Dsg1	**No inhibition** (homophilic): AFM – Heupel, JI 2008 – Walter, Sci Rep 2017 (heterophilic Dsc3): AFM – Spindler, JBC 2009 **Inhibition** (heterophilic Dsc1): beads – Ishii, JID 2020 **No inhibition** (homophilic): AFM – Walter, SR 2017	No Data	No Data	No Data
Dsg3	**Inhibition** (homophilic): AFM – Heupel, JI 2008 – Heupel, JBC 2009 – Spindler, JCI 2013 – Walter, SR 2017 **Inhibition** (heterophilic Dsc3): beads – Ishii, JID 2020	**Inhibition** (homo/heterophilic): AFM – Vielmuth, JID 2015	**Inhibition** No Data	No Data
**PF**	***In vitro*** **(cell-free)**	***In vitro*** **(cultured cells)**	***In vivo*** **(mice)**	***Ex vivo*** **(human)**
Dsg1	**No inhibition** (homophilic): AFM – Waschke, JCI 2005 **Inhibition** (heterophilic Dsc1): – Ishii, JID 2020 **No inhibition** (homophilic): AFM – Walter, SR 2017	**No inhibition** (homo/heterophilic): beads – Waschke, JCI 2005 **No inhibition** (homo/heterophilic): AFM – Vielmuth, Frontiers Immunol 2018	No Data	No Data

**Table 2:** Studies on signalling pathways in pemphigus.

**PV**	**Activation**	**Pathogenic**	**Pathogenic**	**Pathogenic**
		***In vitro* (cultured cells)**	***In vivo* (mice)**	***Ex vivo* (human)**
p38 MAPK	– Berkowitz, JBC 2005 – Berkowitz, PNAS 2006 – Chernyavsky, JBC 2007 – Berkowitz, JID 2008 – Lee, JBC 2009 – Marchenko, JBC 2010 – Spindler, JI 2010 – Mao, JBC 2011 – Bektas, JBC 2013 – Spindler JCI 2013 – Mao, JID 2014 – Spindler, JID 2014 – Hariton, ED 2017 – Walter, Sci Rep 2017 – Vielmuth, FI 2018 – Jin, BA 2021	– Berkowitz, JBC 2005 – Chernyavsky, JBC 2007 – Lee, JBC 2009 – Mao, JBC 2011 – Bektas, JBC 2013 – Spindler JCI 2013 – Mao, JID 2014 – Rötzer, JBC 2014 – Spindler, JID 2014 – Walter, Sci Rep 2017 – Vielmuth, FI 2018 – Burmester, BJP 2020	– Berkowitz, PNAS 2006 – Spindler JCI 2013 – Mao, JID 2014	– Saito, PO 2012 – Egu, BJD 2017 – Burmester, BJP 2020
MK2	– Mao, JID 2014 – Egu, BJD 2019	– Mao, JID 2014	– Mao, JID 2014	No data
HSP25/27	– Berkowitz, JBC 2005 – Berkowitz, JID 2008 – Bektas, JBC 2013 – Mao, JID 2014	No data	No data	No data
RhoA	– Waschke, JCB 2006 – Jin, BA 2021	– Waschke, JCB 2006 – Spindler, AJP 2007 – Gliem, AJP 2010 – Jin, BA 2021	No data	– Waschke, JCB 2006
Adducin	– Rötzer, JBC 2014	No data	No data	No data
Ca^2+^	– Esaki, JID 1995 – Seishima, JID 1995 – Rötzer, JBC 2014 – Walter, Sci Rep 2017 – Schmitt, BJD 2021	– Esaki, JID 1995 – Arredondo, AJP 2005 – Rötzer, JBC 2014 – Walter, FI 2019 – Schmitt, BJD 2021	No data	– Schmitt, BJD 2021
PI4K	No data	– Schmitt, BJD 2021	No data	No data
PLC	– Seishima, JID 1995 – Schmitt, BJD 2021	– Esaki, JID 1995 – Schmitt, BJD 2021	– Sanchez-Carpintero, BJD 2004	– Schmitt, BJD 2021
IP3R	No Data	– Schmitt, BJD 2021	No data	– Schmitt, BJD 2021
CRAC	No Data	– Schmitt, BJD 2021	No data	No data
PKC	– Osada, JID 1997 – Kitajima, JID 1999 – Rötzer, JBC 2014	– Kitajima, JID 1999 – Cirillo, J Cell P 2010 – Spindler, AJP 2011 – Walter, Sci Rep 2017	– Sanchez-Carpintero, BJD 2004 – Spindler, AJP 2011	– Egu, Frontiers 2019
cAMP/PKA	– Spindler, JI 2010 – Walter, SR 2017	– Spindler, JI 2010	– Spindler, JI 2010	– Meier, 2020 FI
Src	– Chernyavsky, JBC 2007 – Pretel, ED 2009 – Marchenko, JBC 2010 – Gil, ED 2012 – Tsang, JP 2012 – Walter, Sci Rep 2017 – Kugelmann, FI 2019	– Chernyavsky, JBC 2007 – Cirillo, AI 2014 – Walter, SR 2017 – Kugelmann, FI 2019	– Pretel, ED 2009 – Gil, ED2012	Not protective
ADAM10	– Ivars, BJD 2020	– Ivars, BJD 2020	No data	No data
MMPs	– Grando, JDS 1992 – Cirillo, JCP 2007	No data	No data	No data
EGFR	– Frusić-Zlotkin, AI 2006 – Chernyavsky, JBC 2007 – Pretel, ED 2009 – Marchenko, JBC 2010 – Bektas, JBC 2013	– Frusić-Zlotkin, AI 2006 – Bektas, JBC 2013 – Sayar, ED 2014 – Walter, FI 2019	– Pretel, ED 2009	No data
	– Sayar, ED 2014 – Walter, FI 2019			
PI3K	No data	– Burmester, BJP 2020	No data	– Burmester, BJP 2020
PDK1	No data	– Burmester, BJP 2020	No data	No data
mTor	– Pretel, ED 2009 – Gil, ED2012	No data	– Pretel, ED 2009	No data
FAK	– Gil, ED2012	No data	– Gil, ED2012	No data
nNOS	No data	No data	– Espana, ED 2013	No data
Casp3	– Arredondo, AJM 2005 – Frusić-Zlotkin, AI 2006 – Dusek, JBC 2006 – Lee, JBC 2009 – Marchenko, JBC 2010 – Pacheco-Tovar, AD 2011 – Gil, ED2012 – Luyet, PO 2015 – Hariton, ED 2017	– Arredondo, AJM 2005 – Frusić-Zlotkin, AI 2006 – Marchenko, JBC 2010 – Pacheco-Tovar, AD 2011 – Luyet, PO 2015 – Schmitt, AJPhysiol 2009	– Pretel, ED 2009 – Hariton, ED 2017	No data
Casp8	– Puviani, JID 2003 – Arredondo, AJM 2005 – Marchenko, JBC 2010 – Lotti, FI 2018 – Sanath, JOMP 2018	– Puviani, JID 2003 – Arredondo, AJM 2005 – Marchenko, JBC 2010	– Pretel, ED 2009	No data
Casp9	– Marchenko, JBC 2010 – Gil, ED2012	– Marchenko, JBC 2010	– Pretel, ED 2009	No data
c-Myc	– Williamson, EMBOJ 2006 – Williamson, JID 2007	No data	No data	No data
JNK	– Marchenko, JBC 2010	No data	No data	No data
c-Jun	– Frusić-Zlotkin, AI 2006	No data	No data	No data
PG	No data	– Spindler, JID 2014	No data	No data
ERK/MEK	– Frusić-Zlotkin, AI 2006 – Bektas, JBC 2013 – Rötzer, JBC 2014 – Walter, Sci Rep 2017 – Radeva, Frontiers 2019 – Walter, FI 2019	– Frusić-Zlotkin, AI 2006 – Walter, Sci Rep 2017 – Radeva, Frontiers 2019 – Burmester, BJP 2020	No data	– Egu, FI 2019 – Burmester, BJP 2020
Calmodulin	No data	No data	– Sanchez-Carpintero, BJD 2004	No data
Calpain	– Arredondo, AJM 2005	No data	No data	No data
CytC	– Marchenko, JBC 2010		No data	No data
CETP	No data	– Burmester, BJP 2020	No data	No data
FAS	– Puviani, JID 2003 – Wang, Appt 2004 – Arredondo, AJM 2005 – Lotti, FI 2018	– Puviani, JID 2003 – Wang, Appt 2004 – Marchenko, JBC 2010 – Lotti, FI 2018	No data	No data
FLIP	– Arredondo, AJM 2005	No data	No data	No data
PLK1	No data	– Burmester, BJP 2020	No data	No data
TrkA	No data	– Burmester, BJP 2020	No data	– Burmester, BJP 2020
VEGFR2	No data	– Burmester, BJP 2020	No data	– Burmester, BJP 2020
**PF**	**Activation**	**Pathogenic**	**Pathogenic**	**Pathogenic**
		***In vitro*** **(cultured cells)**	***In vivo*** **(mice)**	***Ex vivo*** **(human)**
p38 MAPK	– Berkowitz, JID 2008 – Lee, JBC 2009	– Walter, Sci Rep 2017 – Yoshida, JDS 2017	– Berkowitz, AJP 2008 – Lee, JBC 2009	– Yoshida, JDS 2017
	– Walter, Sci Rep 2017 – Vielmuth, FI 2018	– Vielmuth, FI 2018		
HSP25/27	– Berkowitz, AJP 2008 – Berkowitz, JID 2008	No data	No data	No data
RhoA	– Waschke, JCB 2006	– Waschke, JCB 2006 – Spindler, AJP 2007	No data	– Waschke, JCB 2006
Src	– Walter, Sci Rep 2017	– Walter, Sci Rep 2017	No data	No data
c-Myc	– Williamson, EMBOJ 2006 – Williamson, JID 2007	No data	No data	No data
ERK/MEK	– Walter, Sci Rep 2017 – Walter, FI 2019	– Walter, Sci Rep 2017	No data	No data
EGFR	– Sayar, ED 2014 – Walter, FI 2019	– Sayar, ED 2014 – Walter, FI 2019	– Pretel, ED 2009	No data
Casp6	– Li, JI 2009	No data	No data	No data
Ca^2+^	– Seishima, JID 1995 – Walter, Sci Rep 2017 – Walter, FI 2019 – Schmitt, BJD 2021	– Walter, FI 2019 – Schmitt, BJD 2021	No data	No data
PLC	– Seishima, JID 1995	No data	No data	No data
PKC	No data	– Walter, Sci Rep 2017	No data	No data

## Direct Inhibition of Desmoglein Adhesion vs. Cellular Signalling

The cadherins were reported to undergo homo- as well as heterophilic Ca^2+^-dependent interactions *via* their extracellular domains (3). The outermost extracellular domain EC1 domain seems to be most important for Dsg adhesion (1, 27, 162–164). As discussed above, the pathogenic autoantibodies found in pemphigus mostly bind to this domain (27, 162, 165, 166). At first glance, it appears obvious that the binding of a large protein like an antibody to this region would cause steric hindrance by directly inhibiting the Dsg binding and thus cell adhesion. For Dsg3 it was possible to show the occurrence of such a direct inhibition by PV-IgG *in vitro*, both under cell-free conditions and for adhesion to the cell-surface (151, 167) (Table 1). A PV-mouse-model did also show possible indications of direct inhibition of Dsg3 adhesion (165). The finding that the monoclonal anti-Dsg3-antibody AK23, which causes a PV-like phenotype in mice (112), also causes direct inhibition of homophilic Dsg3 interaction under cell-free conditions supports this hypothesis (152, 168). Homophilic Dsc3 and heterophilic Dsc3-Dsg1 were reported to be important for adhesion in keratinocytes as well. Direct inhibition of these interactions greatly reduced the adhesive strength (64). However, another study reported heterophilic interactions between Dsg1 and Dsc1 and Dsg3 and Dsc3 to be the fundamental adhesive unit of desmosomes. In a cell-free assay, mixtures of Dsg1 and Dsc1 or Dsg3 and Dsc3 ectodomains formed large aggregates while Dsg1 or Dsg3 as well as Dsc1 or Dsc3 alone only formed minor to no aggregates. Autoantibodies where effective to directly inhibit binding of Dsg1 to Dsc1 and Dsg3 to Dsc3 beads (169). Direct inhibition has thus to be considered to play a role in PV pathology (150, 167, 170). However, direct inhibition alone is not adequate to cause a complete loss of keratinocyte adhesion. This is highlighted by the fact that impairing cellular signalling but not direct inhibition by incubating the cells at 4 °C did prevent PV-IgG induced loss of cohesion (126). Moreover, inhibition of p38MAPK rescued cell adhesion although direct inhibition of Dsg3 binding by PV-IgG was still detectable which demonstrates that modulation of autoantibody-induced signalling is sufficient to outbalace effects of steric hindrance (137). For PF, the intracellular signalling seems to be even more important since no direct inhibition of Dsg1 interaction was observed in several studies (151, 152, 168, 171).

Over time, several signalling pathways have been identified to be essential for the pathology of pemphigus (59, 152, 171–173) (Table 2). The focus of current research is to integrate all the different pathways and to define their role in pemphigus pathogenesis. Especially, the chronology of many steps is not clear at present.

## The Pathology of Patients' Lesions Identifies the Underlying Mechanisms Interfering With Desmosome Turn-Over

Microscopic evaluation of skin lesions in pemphigus revealed that the pathogenic mechanisms involved cannot be simple. Rather, the different clinical phenotypes in PV and PF are defined by characteristic histology and ultrastructural alterations of desmosomes, which only can be explained by severe impairment of desmosome turn-over, have been established as hallmarks of the disease.

In PV, deep epidermal blisters and mucosal erosions typically separate supra-basal keratinocytes in the blister roof from tomb-stone-like cells of the basal layer in the blister bottom (174, 175) (Figure 1). In contrast, superficial erosions in PF tear of granular and apical spinous cell layers. Since Dsg3 is the predominant desmosomal cadherin in oral mucosa whereas Dsg1 is sparsely expressed, it is conceivable why in PF mucosal erosions usually are absent. Similarly, because in the granular layer Dsg1 and Dsc1 are the main desmosomal cadherins expressed, superficial blistering in PF can be explained by the Dsg compensation hypothesis (176). Supporting this, it was reported, that in neonatal mouse skin, the more ubiquitous distribution of Dsg3 without a distinct enrichtment of Dsg1 in the upper epidermal layers provided a protective effect against PF-IgG (16). However, the morphology of epidermal lesions in PV does not reflect the distribution patterns of Dsg1 and Dsg3 which in human skin broadly overlap. Thus, desmoglein compensation cannot explain a sharp supra-basal cleavage plain as found in PV (3, 150). Also, morphologic alterations typical for apoptosis usually are not present on histologic or ultrastructural levels in patients' lessons demonstrating that apoptotic cell death is not a major cause for acantholysis (154, 177, 178).

It has been shown that Dsg3 clustering in the absence of epidermal blistering correlates with the presence of autoantibodies against Dsg3 in patients with mucosal-dominant PV (149, 178, 179). Similarly, inter-desmosomal widening in PV and PF was present in unaffected epidermis suggesting that these events are not sufficient to cause skin blistering. Rather, epidermal blistering occurred when autoantibodies against Dsg1 were present in PV and PF, the number of desmosomes was reduced, desmosomes were smaller in size and keratin filament retraction from desmosomal plaques was identified by electron microscopy (145, 178, 180). Split desmosomes with partial or complete separation of the two desmosomal plaques from neighbouring cells and double-membrane structures, reflecting engulfment of membrane domains, were detected also. Thus, the best strategy to evaluate the relevance of the different mechanisms causing pemphigus pathology is to correlate mechanisms with the morphologic hallmarks of the disease. Direct inhibition of desmoglein binding by steric hindrance through bound autoantibodies would cause splitting of desmosomes but no alterations of desmosome ultrastructure. Indeed, mechanical shear has been demonstrated to cause split desmosomes (181) which therefore may be caused by steric hindrance. Since split desmosomes mostly were found to be smaller and displayed altered insertion of keratin filaments (15, 178, 181, 182), splitting of desmosomes most likely is a consequence of altered desmosome composition. Moreover, most desmosomes in *ex vivo* human skin models of pemphigus are not split but rather smaller and characterised by less opaque plaques, less dense desmoglea in the desmosomal intercellular space and shorter keratin filaments attached (15, 182). Taken together, the ultrastructural alterations can be explained only by impaired desmosome turn-over (178, 183).

Therefore, the major goal is to decipher which mechanisms shown to be induced by autoantibodies interfere with the distinct phases of desmosome assembly and dissasembly. The different steps of desmosome assembly have been characterised in detail (184). First, Dsg and Dsc molecules together with PG are transported to cell borders with preformed adherens junctions along microtubules and *via* kinesin. At the membrane, desmosomal cadherins associate with lipid rafts *via* Flotillin-1 and 2 which are required for proper desmosome assembly (185, 186). Here, intermediate junctions composed of Dsgs and adherens junction components including E-cadherin, PG and β-catenin are assembled in Src-dependent manner (138, 187, 188). In a next step, Dsg molecules are transferred to DP coupled to keratin filaments (9, 189). Actin binding proteins such as adducing, which is regulated *via* RhoA/Rho kinase, and cortactin are important for directed targeting of Dsg molecules during desmosome assembly (190–193). The assembly of Dsgs with DP is controlled by PKP3 in a Rap1-dependent manner (194), the latter of which is a GTPase activated by cAMP and EPAC1. Inside of desmosomes, Dsg isoforms including Dsg1 and Dsg3 in a Ca^2+^-dependent and most likely homo- and heterophilic manner interact with Dsc1 and Dsc3 *via* their extracellular domains (64, 168, 169, 171, 188, 195).

## Hyperadhesion as a State of Increased Desmosome Stability

Finally, the desmosomal components are locked inside of desmosomes by signalling mechanisms which control the anchorage of DP to keratin filaments and thereby induce a hyperadhesive state (196). This indicates that once a desmosome is established it is remarkably stable but the protein exchange of desmosomal components is regulated on the molecular level resulting in low half-life of desmosomal components (147, 196, 197). This paradigm allows desmosome disassembly when the net exchange by molecules diffusing out of desmosomes outbalances the assembly process as described above in processes like wound healing and migration (198–200). Also, this explains why uptake of entire desmosomes in double-membrane structures as observed under pathologic conditions in pemphigus *ex vivo* models is rarely observed in intact epidermis (182).

Interestingly, under hyperadhesive conditions the binding properties of desmogleins change dramatically. For instance, in hyperdahesive keratinocytes oligomerization of Dsg3 but not Dsg1 is enhanced and the Ca^2+^-dependency of desmosome integrity is abolished (196, 198, 201–203). Rather, if trans-interaction between Dsg3 molecules was maintained desmosomes were preserved under Ca^2+^-depletion although the ordered array of the Dsg3 extracellular domains in desmosomes was lost (196). This indicates that Ca^2+^-dependency of Dsg3 interaction may get lost when adhesion molecules and plaque proteins in desmosomes are locked by cytoskeletal anchorage. Moreover, since it was shown that Dsg1 interaction can be rescued after re-substitution of Ca^2+^ whereas Dsg3 cadherin order was not re-established (171, 196, 204), it is also possible that the roles of Dsg1 and Dsg3 in hyperadhesion are different. This can be concluded from the observation that in intact human epidermis, in which the majority of desmosomes is assumed to be hyperadhesive (198, 201, 202, 205), immunostaining of Dsg1 but not of Dsg3 was drastically altered by Ca^2+^ depletion indicating that Dsg3 in the epidermis indeed loses its Ca^2+^ dependency (206). In line with this, the molecular binding strength of Dsg3 but not of Dsg1 was increased when keratinocytes were hyperadhesive.

As the underlying molecular mechanism regulating hyperadhesion, PKC-mediated phosphorylation of DP under control of PKP1 has been shown to result in decreased cytoskeletal anchorage and thus to revert the hyperadhesive state in order to allow desmosome reorganisation (196, 197, 199–201, 207, 208). To allow this adaption, PKC was shown to be sequestered to desmosomes *via* the adaptor-protein RACK1 in DP-dependent manner and is regulated by keratins (170, 208, 209). However, since in cultured cardiomyocytes and cardiac slice cultures besides inhibition of PKC a hyperadhesive state can also be induced by PKA activation and p38MAPK inhibition, it is likely that other signalling mechanisms involved in the assembly of the desmosomal plaque and its anchorage to keratin filaments are also important for hyperadhesion (210–212). Other targets besides DP appear to be relevant as well because hyperadhesion can be abolished when expression of desmosomal components such as Dsg3 or PKP1 and PKP3 is disturbed (203, 213), which is also similar to cardiomyocytes where depletion of Dsg2, PG, or PKP2 abrogated hyperadhesion (214).

An open question, which also has relevance to identify the central molecular mechanisms impairing desmosome turn-over in pemphigus remains: whether the hyper-adhesive state can be recognised on the level of desmosome ultrastructure. It has been proposed that a dense midline in the desmosome intercellular space where extracellular domains of neighbouring cells trans-interact would be a hallmark of hyperadhesion (198). Indeed, in intact epidermis most desmosomes exhibit dense midlines which is not the case in the vicinity to epidermal blisters or where the desmosome ultrastructure is altered in *ex vivo* human pemphigus skin models (182, 215). However, in cultured keratinocytes under hyperadhesive conditions desmosomes were lacking midlines completely (196). This indicates that the hyperadhesive state cannot be concluded for a specific desmosome from its ultrastructure in cultured cells. On the other hand it can be assumed that in the epidermis desmosomes with altered morphology and lacking midlines most likely are not hyperadhesive and thus PKC is likely to participate in pemphigus pathology by reverting hyperadhesion of desmosomes.

## Autoantibodies Against Dsg1 AND Dsg3 Induce Specific Signalling Pathways in Pemphigus

**Figure 2:**
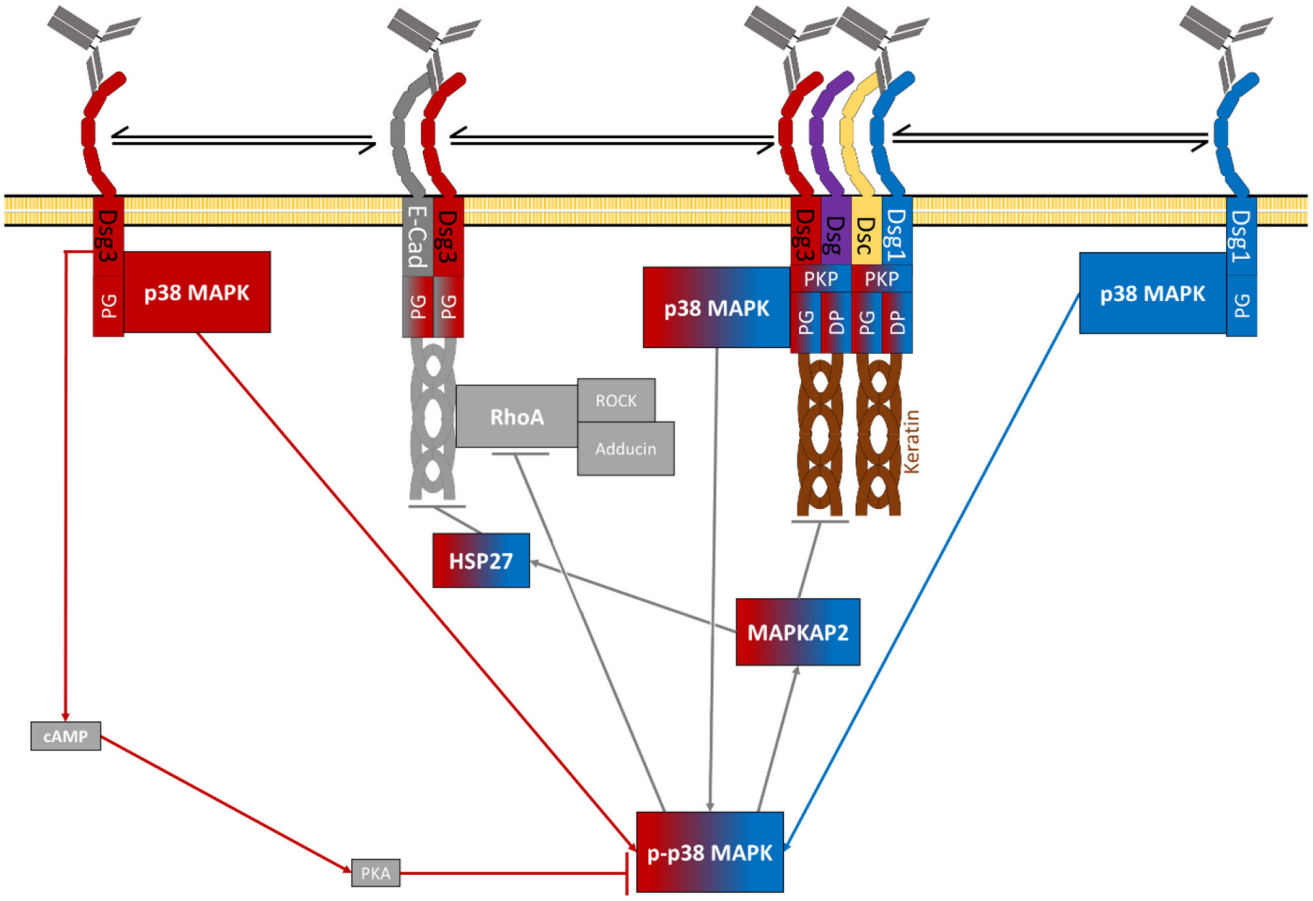
Schematic depiction of p38MAPK-dependent signalling in pemphigus (grey RhoA and cAMP steps represent potential rescue pathways).

**Figure 3:**
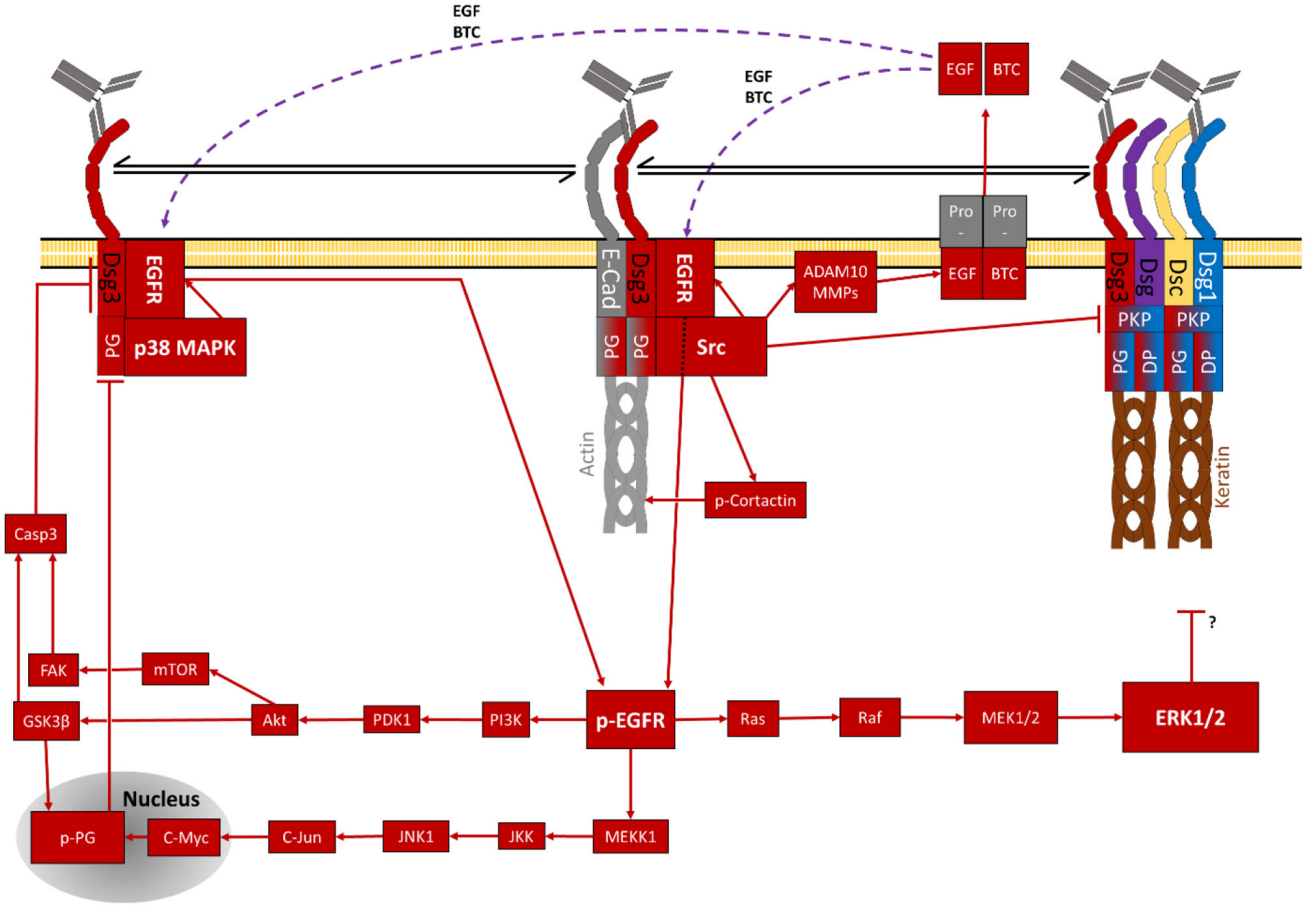
Schematic depiction of EGFR-dependent signalling pathways (purple lines indicate diffusion of extracellular second messengers).

**Figure 4:**
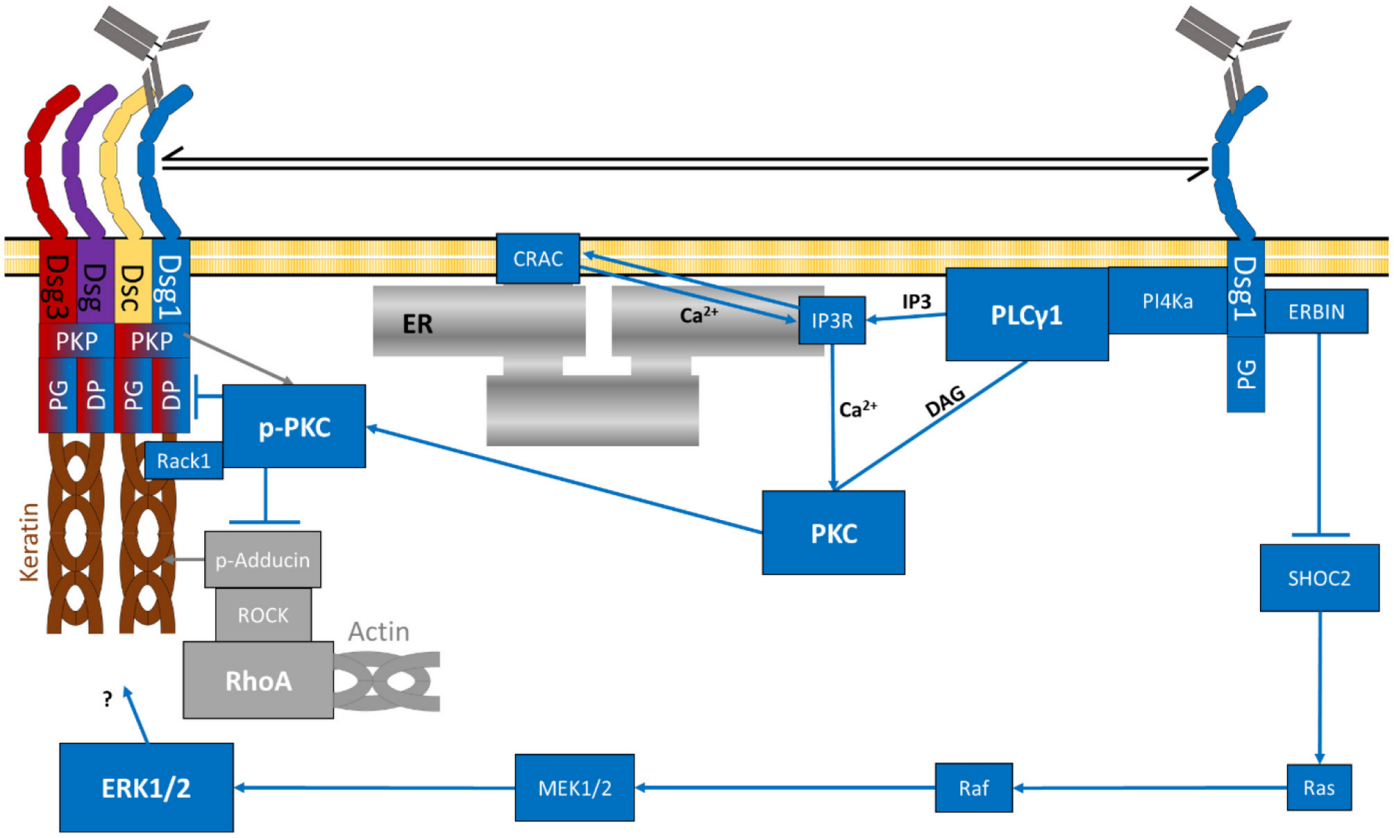
Schematic depiction of specifically Dsg1-dependent signalling mechanisms (grey RhoA complex represents a potential rescue pathway). The specific target of Erk1/2-Elk1 is not known.

**Figure 5:**
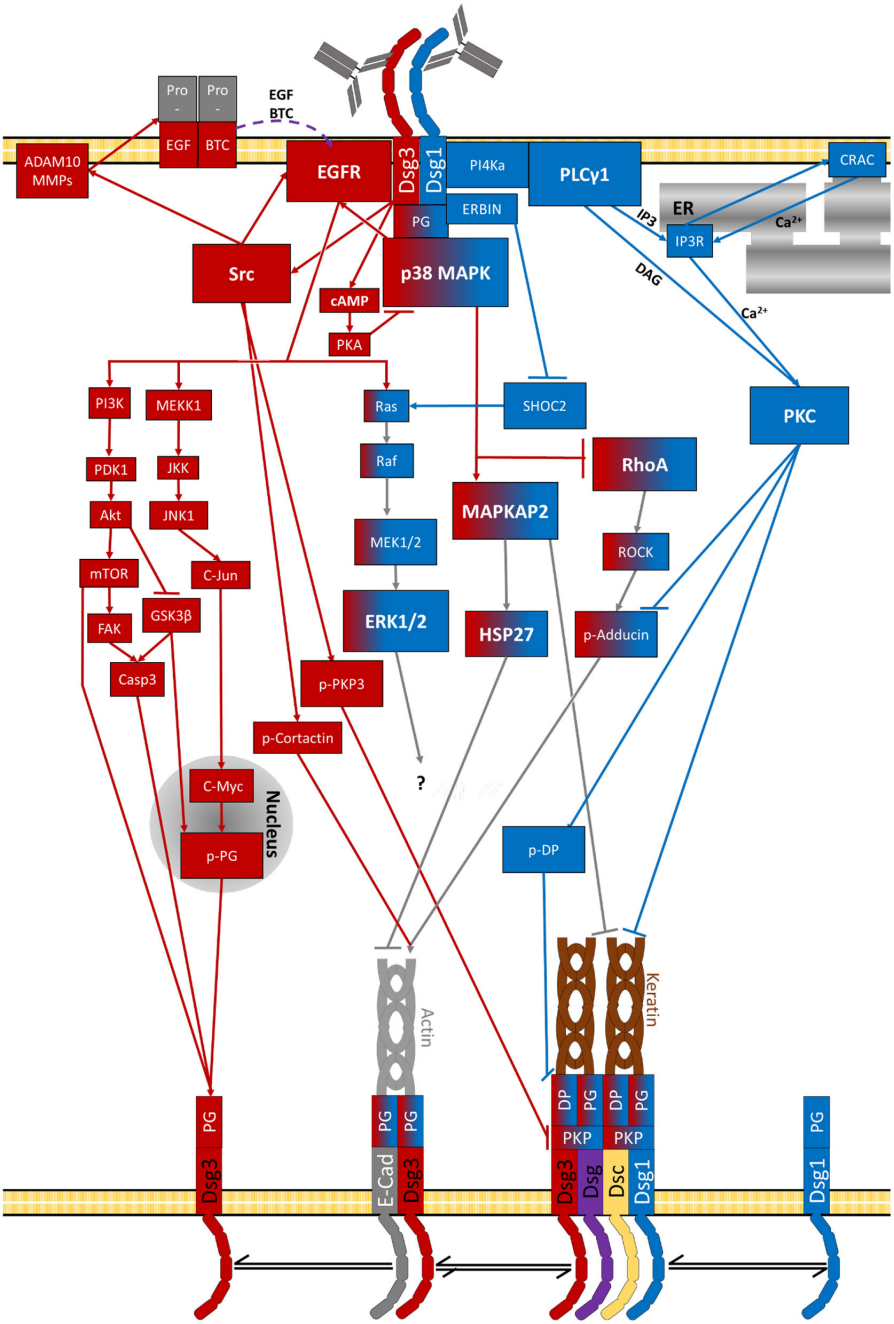
Signalling pathways activated by pemphigus-IgG (purple lines indicate diffusion of extracellular second messengers). The specific target of Erk1/2-Elk1 is not known.

As summarised above a vast set of signalling pathways has been associated with pemphigus pathogenesis (Figures 2–**5** and Table 2). Before we try to allocate the different mechanisms to the steps of desmosome assembly and disassembly, we introduce the concept that the signalling pathways activated by a specific autoantibody are defined by the signalling complexes organised by Dsg1 and Dsg3. It was an important observation that signalling responses in keratinocytes are not a consequence of loss of cell adhesion but rather signalling molecules are associated with Dsg isoforms. This was shown first for Dsg3, which was found to form a complex with activated p38MAPK, a process enhanced by pemphigus autoantibodies (216). This adhesion receptor was regulated in adhesion-dependent manner because reduced Dsg3 adhesion by specific autoantibodies or peptides or tryptophan caused p38MAPK activation whereas stabilisation of Dsg3 binding by cross-linking peptides reduced p38MAPK activity (216, 217). This process is regulated by PG because depletion of PG also caused p38MAPK activation. In contrast, depletion of DP or PKP1 and PKP3, which also cause a profound loss of keratinocyte adhesion, did not enhance p38MAPK activity (203, 218, 219).

The signalling complexes organised by Dsg molecules appear to be both isoform- and cell type-specific because Dsg2 in contrast to Dsg3 and Dsg1 does not associate with p38MAPK (14, 220, 221). Nevertheless, in enterocytes Dsg2 regulates p38MAPK activity (222). In contrast, Dsg2 has been shown to bind and to control EGFR in enterocytes in fashion similar to Dsg3 in keratinocytes (187, 223).

All of this led us to propose that Dsg molecules form cell-type specific signalling complexes which serve as adhesion-dependent signalling receptors and regulate complex functions such as keratinocyte migration and wound healing (59, 224). Most recently, we observed that Dsg1 also forms signalling complexes which are at least in part different to Dsg3. The majority of signalling molecules involved in pemphigus pathogenesis analysed so far including p38MAPK, PLC, and PKC were associated with both Dsg1 and Dsg3. However, PI4K acting upstream of the PLC/Ca^2+^/PKC pathway was found exclusively to interact with Dsg1 (220). The fact that p38MAPK binds to both Dsg1 and Dsg3 is compatible with the observation that blister formation *in vivo* caused by PV-IgG and PF-IgG is blocked by inhibition of p38MAPK (225, 226) and that both PV-IgG and PF-IgG reduced activity of RhoA in p38MAPK-dependent manner (127, 227).

Moreover, these findings are in line with the observation that autoantibody fractions including antibodies targeting Dsg1 in PV and PF induce a different signalling response compared to antibodies against Dsg3. Most striking, PLC activation and Ca^2+^ influx was found only when Dsg1 autoantibodies were present in PV-IgG and PF-IgG fractions but not induced by mucosal-dominant PV-IgG or AK23 (152, 220, 228). Similarly, ERK activation was detectable after incubation with PF-IgG and PV-IgG only when Dsg1 antibodies were present and similar to Ca^2+^ influx activation was preserved in Dsg3-deficient keratinocytes. In contrast, EGFR activation was found to be dependent on Src and caused by AK23 and mucosal-dominant PV-IgG and thus in the absence of autoantibodies against Dsg1 (152, 228). Similarly, Src- and ADAM10-dependent EGFR activation has been found in response to PV-IgG including autoantibodies against Dsg1/3 whereas blister formation with additional autoantibodies against Dsc2/3 was ADAM10-independent (66).

For PKC it is not clear whether the different isoforms are regulated *via* Dsg1 or Dsg3. PKC was found to associate with both Dsg1 and Dsg3 and inhibition of PKC ameliorated loss of cell adhesion in response to PF-IgG and PV-IgG independent of the presence of autoantibodies against Dsg1 (152, 220). Also, PKC was found to be involved in Dsg3 depletion *in vivo* (127). On the other hand, at least for Ca^2+^-dependent PKC isoforms activation *via* autoantibodies against Dsg1 can be assumed (220).

Taken together, the studies on composition of signalling complexes on one hand and evaluation of signalling pathways on the other hand suggest that besides p38MAPK activation autoantibodies against Dsg3 in PV cause SRC-dependent EGFR activation whereas autoantibodies against Dsg1 in PV and PF activate both ERK and the PI4K/PLC/Ca^2+^ pathway. The hypothesis that the signalling pattern orchestrated by Dsg1 and Dsg3 in pemphigus are to some extent different also helps to explain why blister formation in pemphigus requires antibodies against Dsg1. In this line of thoughts, some pathologic effects such as clustering and depletion of Dsg3 molecules in unaffected skin of mucosal-dominant PV patients (149, 179) can be induced by signalling pathways such as p38MAPK, Src/EGFR and PKC. However, clustering of Dsg1 as well as shrinkage and loss of desmosomes which finally cause epidermal blistering (149, 179, 182) require ERK and PLC/Ca^2+^ signalling in addition to p38MAPK. This third line of evidence that the relative contribution of various signalling pathways for skin blistering in pemphigus is different comes from studies in human skin *ex vivo* where inhibition of p38MAPK, ERK, and PLC activity as well as of Ca^2+^ influx were sufficient to abrogate skin blistering whereas inhibition of Src and PKC was not (182, 191, 215, 220, 229).

Another important aspect is that inhibition of p38MAPK is effective to abolish PV-IgG-induced epidermal blistering in *ex vivo* human skin models whereas antibodies against Dsg3 such as AK23 or in mucosal-dominant PV-IgG fractions are not sufficient to induce skin blistering, although they activate p38MAPK (152, 182). This suggests that p38MAPK for epidermal blistering is required but not sufficient. In a mucosa model, inhibition of p38MAPK did not block blistering although AK23 in mucosa activated p38MAPK indicating that the relevance of signalling pathways in pemphigus is also tissue-specific (15). All these results indicate that the clinical phenotype of PV with mucosal erosions and deep epidermal blistering on one hand and PF characterised by superficial acantholysis on the other hand are unlikely to be caused by different expression pattern of Dsg3 and Dsg1 alone as suggested by the Dsg compensation hypothesis. Rather, the mechanisms regulating cell adhesion in mucosa and epidermal layers appear to be different and to be regulated by Dsg3 and Dsg1 (3, 150).

Finally, it has to be noted that the observation of Dsg3 and Dsg1 to regulate keratinocyte adhesion and function *via* different mechanisms is also in line with the different phenotypes of mouse models deficient for these molecules. Dsg3-deficient mice besides hair-loss display rather mild erosions in the skin with PV-typical histology, which have been observed to spontaneously heal within several days, but also suffer from eye involvement in conjunctiva and cornea (56, 119, 224). The phenotype may be explained by the observation that in both affected and unaffected skin p38MAPK activity was up-regulated, which was found to be required for wound healing (224). This shows that Dsg3 supports epidermal integrity at least in part *via* regulation of signalling pathways controlling cell adhesion. In contrast, Dsg1-deficient mice lose their superficial epidermis during birth and all die within 24 h due to complete loss of epidermal barrier properties (125). Because TJ morphology was altered in heterozygous mice, it is likely that the function of Dsg1 *via* Erbin to control EGFR-mediated TJ assembly in the superficial epidermis is involved also (230–232) (Figures 2–**5**).

## The Central Role of p38MAPK to Regulate Desmosome Turn-Over

For some signalling pathways the role in pemphigus pathogenesis and the mechanisms underlying loss of desmosomal adhesion became clearer during the last two decades.

An important molecule contributing to pathogenesis of pemphigus is p38MAPK, a protein involved mostly in the reaction to external stressors, including oxidative (233, 234), thermal and chemical stress (234, 235) but can also be activated by growth factors (236). Upon activation by phosphorylation at threonine residue 180 and tyrosine 182 (225, 237–239) p38MAPK activates other kinases further downstream and finally impacts cell growth, differentiation (240), migration as well as wound healing (224) possibly also modulating gene expression (235) and was also reported to directly phosphorylate keratins in keratinocytes and enterocytes (241–243).

p38MAPK is activated by PV-IgG (225, 237) as well by PF -IgG (226) in keratinocytes *in vitro* and in mouse skin *in vivo*. p38MAPK is associated with both intra- as well as extradesmosomal Dsg3 (187) and is phosphorylated in a protein complex with Dsg3 and PG (216). p38MAPK then *via* MAPKAPK2 (56, 244) activates HSP27 (225, 237, 245). In skin biopsies of PV and PF patients, activation of p38MAPK (238), MAKAPK2 (244) as well as HSP27 (238) were confirmed also. HSP27 regulates both actin (236) as well as intermediary filament (246) dynamic restructuring. Moreover, p38MAPK is associated with the keratin cytoskeleton (151, 161, 216, 237).

To delineate the role of p38MAPK in pemphigus pathogenesis it was shown that specific inhibition of p38MAPK or its downstream target MAPKAPK2 are sufficient to reduce loss of adhesion in keratinocytes *in vitro* (221, 247), depletion of both extra- and intradesmosomal Dsg3 (132, 233, 244) from both the soluble and insoluble fractions (132) and the activation of and reorganisation of the cytoskeleton by HSP27 (237) upon PV-IgG treatment. In murine skin *in vivo*, p38MAPK inhibition was sufficient to prevent skin blistering and activation of HSP25, the murine analogue to human HSP27, in the skin (225, 226) upon PV- or PF-IgG treatment. In human skin *ex-vivo*, inhibition of p38MAPK ameliorated the pathogenic effects of PV-IgG, including skin blistering, Dsg3 fragmentation, reduction of desmosome number and size, membrane detachment and keratin retraction (182). In contrast, inhibition of p38MAPK was protective against Dsg1 clustering but did not prevent acantholysis induced by PF-IgG (113).

However, it is currently unclear how exactly p38MAPK is activated after antibody binding to Dsg1 and Dsg3. One study reported activation with two peaks in activity at different points in time, which was different *in vivo* vs. *in vitro* as well as between PV- and PF-IgG. It was proposed that both peaks would most likely cause different cellular responses (248). In other experiments, different peaking patterns were reported (249) or experiments were only performed at specific time points (233) making it impossible to directly compare many of these results (Figures 2, **5**).

## Rho A/Adducin-Mediated Desmosome Assembly

One pathway by which p38MAPK could deplete Dsg3 from intra- and extradesmosomal pools is interference with RhoA/adducing-mediated assembly of desmosomes. Reorganisation of the actin cytoskeleton was observed upon treatment with both PV-IgG and PF-IgG (171, 237). RhoA was shown to induce reorganisation of the actin cytoskeleton (157) and is inactivated following PV-IgG and PF-IgG treatment (227). This mechanism appears to be important in pemphigus because pharmacologic activation of RhoA inhibited epidermal blistering in response to PV-IgG and PF-IgG in human skin *ex vivo* and inactivation of Rho-GTPases induced epidermal splitting similar to autoantibodies (227, 250). It was demonstrated that inhibition of p38MAPK abolished RhoA inactivation after PV-IgG treatment identifying RhoA as another downstream target of p38MAPK (127, 227). PKP2 was shown to prevent the localisation of RhoA to cellular interfaces having similar effects as RhoA inactivation (190). RhoA activity was furthermore linked to possible apoptotic signalling in pemphigus (251). The effects of RhoA inactivation include reduced incorporation of DP into the desmosomal plaque (190), reduced anchorage of the desmosomes to the keratin cytoskeleton (227) and depletion of Dsg3 and Dsg1 finally leading to loss of cell adhesion (250, 252–254).

All these effects can be explained by the fact that RhoA *via* Rho kinase regulates the actin binding protein adducin (192). Adducin is essential for cell adhesion in keratinocytes by controlling the incorporation of Dsg3 into desmosomes (193). Other actin binding proteins such as cortactin, which have been shown to be involved in pemphigus pathogenesis, also bind to extra-desmosomal Dsg3 and thus may participate in desmosome assembly (191). Thus, it is possible that inhibition of desmosome assembly *via* p38MAPK-mediated inactivation of Rho family GTPases and actin binding proteins is a general paradigm in pemphigus pathogenesis (Figures 2, **5**).

## Plakoglobin Acts as Signal Transducer and Transcription Factor in Pemphigus

As described above, PG is essential for the assembly of desmosomes (255–257) and cellular adhesion (8, 256, 258) e.g., as part of the Dsg3-PG complex (187). This complex is later incorporated into the desmosomes and becomes insoluble *via* interaction with Dsg1 (259). It was shown, that PV-IgG impairs the distribution of PG (260–262) and its association with Dsg3 (263). As described this is a result of PG endocytosis (218, 260). It was shown that the composition of the desmosomes and cellular adhesion and keratin filament integrity is largely dependent on PG (218, 261). Loss of PG activated p38MAPK as discussed above (218). However, PG was reported to contribute to both desmosomes and adherents junctions (255, 256) but is also found free at the cell membrane as well as in the cytosole (130) and even in the nucleus (218) of keratinocytes. It is thus most likely displaying an even broader range of functions. The localisation of PG is regulated by phosphorylation *via* GSK3β and thus inhibitors of this pathway were shown to be protective against PV-IgG-induced skin blistering *in vivo* (264). By its relocalization to the nucleus, PG also regulates gene expression in pemphigus patients. This role is distinct from its function as a plaque protein (218). PG increases expression of urokinase-type plasminogen-activator receptor (uPAR) at the cell membrane. uPAR was described to be involved in wound healing and cellular reorganisation at the cell membrane (262). Nuclear PG also increased expression of the proto-oncogene c-Myc, which ultimately leads to hyperproliferation, reduced cell adhesion and eventually skin blistering in pemphigus patients (264, 265). This increase of nuclear c-Myc is characteristic for PV and is not observed for other skin diseases including PF, again highlighting that for both variants of pemphigus different signalling pathways are activated. It was observed that c-Myc activity is already increased very early in the pathogenesis and does correlate with disease severity (265). Very recently, inhibitors against the signalling cascade PI3K/PDK1/Akt, which regulate GSK3β, have been shown to be protective against skin blistering *in vivo* and *ex vivo* human skin and also inhibition of the down-stream molecule mTOR was shown to protective in mice (229, 266). Therefore, it is possible that this signalling pathway regulates desmosome turn-over also by mechanisms different from PG (Figures 3, **5**).

## The Src/ADAM10/EGFR Receptor Pathway in Pemphigus

Src activated rapidly and in a Dsg3-dependent manner (267) after PV-IgG treatment (249). Src activity was reported to cause loss of Dsg3 from the membrane, phosphorylation of PKP3, thereby destabilising the desmosomal plaque (268) and to induce keratin retraction (249). These changes cause loss of adhesion *in vitro* (191, 268) and skin blistering in mice *in vivo* (66, 172, 191, 266). In peri-lesional skin of pemphigus patients an increased Src activity was observed. In contrast to that, in lesional skin its activity was notably reduced, indicating an activation of Src before akantholysis (267). However, in human skin Src inhibition was not completely effective to prevent acantholysis caused by PV-IgG fractions including higher anti-Dsg1 IgG concentrations. Because Src was no longer activated after 24 h and Src inhibition was only protective during timeframes when Src was active (191), it can be assumed that Src plays a less central role in pemphigus pathogenesis and likely contributes during the initial phase only when the extradesmosomal Dsg3 is depleted. In addition to its pathogenic role, Src is also essential for the Ca^2+^-dependent incorporation of Dsg3 into desmosomes by stabilising the complex of E-cadherin together with Dsg3, potentially *via* cortactin (187, 191). Thus, Src seems to play an interesting dual role by stimulating desmosome formation under basal conditions but causing desmosome disassembly under pathogenic conditions.

One target molecule of Src in pemphigus pathogenesis appears to be EGFR which was shown to interact with (8, 269) and to be activated *via* Src (228, 249, 270). EGFR inhibition was shown to be protective against effects induced by PV-IgG and PF-IgG *in vitro* and *in vivo* (228, 271–273). EGFR is an important central molecule of a complex network of signalling pathways (269) and can be activated by ligands like the EGF or TGF but also in a ligand-independent manner *via* phosphorylation by p38MAPK and formation of intracellular signalling complexes (269, 270, 272, 274). EGFR was shown to regulate cellular adhesion and inhibiting it's basal activity promotes localisation of PG and DP to the desmosomal plaque and strengthens cellular adhesion (272, 275). Furthermore, it was demonstrated to be involved in pemphigus signalling (249, 272, 273) but not to be crucial because EGFR-independent loss of keratinocyte adhesion after incubation with PV-IgG has been observed as well (217). EGFR activity leads to endocytosis of Dsg3 and PG causing loss of adhesion (272, 273).

EGFR downstream signalling is broad and the exact mechanisms involved in acantholysis are not perfectly clear but may involve PI3K and ERK which recently were associated with pemphigus pathogenesis (152, 215, 229). PI3K, acting *via* PDK1/Akt/mTOR/GSK3β on nuclear localisation of PG may cause hyper-proliferation and loss of adhesion (269, 276, 277). Similarly, the Ras/Raf/MEK/ERK pathway is involved in the regulation of cell proliferation vs. differentiation and causes loss of adhesion as well (228, 230, 269, 278–281). Another possible target is JNK1 impacting cell proliferation and migration (269, 282, 283).

Besides direct activation of EGFR by phosphorylation at Y845, Src is also involved in the release of activating EGFR ligands in response to pemphigus autoantibodies (66, 228). In this context, metalloproteinases (MMPs) are involved which can cleave cellular proteins and thus convert proteins to active forms, release second messengers or cleave and inactive proteins. They are well-known to be involved in the pathology of bullous pemphigoid (284). It was furthermore reported that in PV and bullous pemphigoid levels of serin proteinases activity were increased in patients' serum and blister exudates whereas MMP inhibitors were reduced (285). MMP9 was reported to be significantly upregulated in PV model *in vivo* and *in vitro* (286). Recently, ADAM10 was reported to be directly involved in pemphigus pathogenesis (66). ADAM10 is activated downstream of Src thereby modulating EGFR activation *via* release of EGF and Betacellulin (BTC). Interestingly, the activation of ADAM10 is dependent on the autoantibody profile and restricted to antibodies targeting Dsg1 and Dsg3. In contrast, additional autoantibodies against Dsc2 and 3 triggered a more severe and earlier acantholysis independent of ADAM10 (66) (Figures 3, **5**).

## Dsg1 Regulates EGFR Signalling Towards ERK Which May Be Involved in Pemphigus

Dsg1 is a very important antagonist of EGFR signalling. EGFR promotes cell migration and proliferation in the basal layer of the epidermis. In contrast, Dsg1 expression in superficial epidermis shifts the gears more towards keratinocyte differentiation. This antagonistic role is a result of at least one shared pathway. While EGFR is known to activate ERK1/2, Dsg1 is a known suppressor of this signalling pathway (215, 228, 230, 279–281, 287). This suppression is mediated *via* the Dsg1-Erbin-SHOC2-complex which interferes with EGFR-induced activation of MEK1/2 and ERK1/2 (228, 279–281). Therefore, this pathway represents a very important crossing point between Dsg3- and Dsg1-dependent signalling and a protective role of Dsg1 against signalling induced by anti-Dsg3 autoantibodies in pemphigus can explain why in mucosal PV the epidermis is not affected although Dsg3 distribution is severely altered (149, 178, 179). In this line of thoughts, anti-Dsg1 antibodies in PV would shut off the inherent suppression on ERK *via* Erbin-SHOC2 and thereby activate ERK in the absence of EGFR activation as observed following incubation with PV-IgG and PF-IgG (152, 228). This hypothesis is also in line with the inefficiency to prevent loss of adhesion by EGFR suppression in presence of higher concentrations of anti-Dsg1 autoantibodies, which releases the suppression of Erk1/2 signalling (217) (Figures 3, **5**).

## Dsg1-Mediated Ca^2+^-Signalling is Important For Epidermal Blistering in Pemphigus

Relatively early in pemphigus research, PV-IgG treatment was demonstrated to induce activation of PLC and Ca^2+^-influx *via* IP3R (156, 160). More recently, direct interaction of DSG1 and PI4K (220, 288, 289), an upstream kinase of PLC (290), was reported. For PLC, an interaction with both Dsg1 and Dsg3 was shown (220). Activated PLC generates the second messengers IP3 and DAG (156, 172) which leads to a release of Ca^2+^ from the endoplasmic reticulum (ER) to the cytoplasm (291). Stim1 and Orai1 are two ubiquitously expressed proteins, which together form the so-called CRAC (Ca^2+^-release-activated-channel). Stim 1 is located at the membrane of the ER (292) where it serves as a sensor for the Ca^2+^ concentration. Upon low Ca^2+^, Stim1 contacts Orai1, located at the plasma membrane, and both together from a Ca^2+^-specific channel causing Ca^2+^ influx and replenishing the Ca^2+^ stores of the ER (293–298). In line with this, inhibition of Ca^2+^ signalling *via* inhibition of either PI4K, PLC, IP3R or CRAC was sufficient to ameliorate pathogenic effects of pemphigus autoantibodies *in vitro* and inhibition of PLC and IP3R abrogated skin blistering in human skin *ex vivo* (220).

DAG and Ca^2+^ released by these mechanisms are activators of PKC (195, 299), causing its redistribution to cell contacts (200, 299). PV-IgG was demonstrated to induce PKC activation as well (127, 152, 172, 208, 300). The exact mechanism of action of PKC is yet unknown, however, it was observed that PKC interacts with the keratin filaments (161, 208, 301) and is regulated by PKP1 (207). PKC was furthermore shown to phosphorylate DP which causes destabilisation of the desmosomal plaque (197, 208, 209). Inhibition of PKC reduces the PV-IgG-induced depletion of Dsg3 (127), keratin retraction (208) and loss of cell adhesion (127, 152) *in vitro* and *in vivo* (127, 172) and skin blistering in mice *in vivo* (127, 172). PKC thus clearly plays a role in pemphigus pathogenesis. However, since it was reported that inhibition of PKC was not sufficient to prevent skin blistering in human skin *ex vivo* (215), PKC is probably not the only downstream target of the PLC/Ca^2+^ pathway. Furthermore, it needs to be noted that there are three types of PKCs with various isoforms, activated by different sets of second messengers. More specific, DAG and cytoplasmic Ca^2+^ activate Ca^2+^-dependent PKC (cPKC), DAG activates novel PKC (nPKC) and translocation activates atypical PKC (aPKC) isoforms, respectively (299). At least cPKCs and nPKCs are downstream targets of PLC and thus may be activated in response to autoantibodies in pemphigus (Figures 4, 5).

## Pre-Apoptotic Caspase Signalling Reducing Dsg1 and Dsg3 in Pemphigus

Several receptor-induced signalling cascades can initiate signalling associated with apoptosis. The caspase protein family plays a central role for this signalling. Caspases cleave certain cellular proteins (154) causing morphologic changes such as condensation of the nucleus, fragmentation of the DNA, cell shrinkage (302) and degradation of the cytoskeleton (303, 304). Amongst the proteins which are cleaved by caspases are the desmosomal cadherins and the plaque proteins causing degradation of desmosomes and cytoskeleton (305). The possibility that these apoptotic pathways can be active in pemphigus was demonstrated in several studies (79, 154, 302, 306–310). Apoptosis can occur in the skin (307, 309, 311, 312) as well as the mucosa (308) of pemphigus patients. However, in acantholytic areas apoptotic cell death is not typically observed (154, 177, 178).

It was shown that PV-IgG increases the activity of pro-apoptotic and decreases that of anti-apoptotic proteins in keratinocytes *in vitro* (309, 311, 313) and mice *in vivo* (266, 306, 307). Caspase3 seems to be the most relevant in this context (306, 307, 314). Caspase signalling may be activated by PV-IgG in several ways. Increased amounts of soluble Fas receptor ligands were found in pemphigus patients' sera and in mice after injection of PV-IgG. These induce activation of caspase8 which causes cleavage of Dsg molecules. Inhibition of Fas receptors and depletion of soluble Fas ligand were also shown to be protective in keratinocytes *in vitro* and mouse skin *in vivo* (309, 315). Activation *via* caspase8 was reported as well (316). In some patients anti-mitochondrial autoantibodies were found and it was shown that they can penetrate into mitochondria. These antibodies were reported to cause JNK activation and *via* caspases may induce degradation of Dsg3 (317, 318). Similarly, Dsg1 was also identified as a caspase3 target (319). The activation *via* c-Jun is also supported by the observation that inhibition of p38MAPK signalling, which causes c-Jun activation *via* EGFR, was sufficient to block caspase3 activation after PV-IgG treatment (248). Furthermore, EGFR was reported to induce caspase signalling and apoptosis (273). Thus, signalling by antibodies against Dsg3 may use this Src/EGFR signalling axis to activate caspases *via* PI3K/PDK1/Akt/GSK3β and nNOS/mTOR/FAK suggestive because inhibitors against this pathway have been shown to be protective against skin blistering (229, 266, 320, 321).

Moreover, caspase signalling could also be induced *via* the Ca^2+^ pathway. In this context, a mechanism was described which links the mitochondrial Ca^2+^ specific channel VDAC1 to IP3R *via* so-called mitochondria-associated membranes (MAM). The resulting Ca^2+^ flux was reported to lead to mitochondrial swelling and induction of apoptosis-related signals (322–326). Caspase3 activity can further be linked to p38MAPK, c-Myc (318), and RhoA activity (251). In line with this, several studies demonstrated that activation of p38MAPK, cell dissociation, and keratin retraction in keratinocytes *in vitro* (318) and skin blistering in mice *in vivo* (79, 266, 306, 307, 318) were significantly ameliorated under pharmacological inhibition of caspase activity.

However, the relevance of apoptosis for the pathology of pemphigus is controversly discussed and often attributed to be a secondary effect caused by acantholysis (111, 154). It was demonstrated that acantholysis takes place independent of apoptosis in keratinocytes *in vitro* (154, 318, 327, 328) as well as in pemphigus patient skin (154, 177, 318, 328). One group reported a slight increase of caspase3 *in vitro* and *in vivo* before the appearance of lesions but without any other sings of apoptosis (318). Taken together, all studies indicate a possible role of caspases for acantholysis independent of apoptotic cell death (306, 318). Since it was already found that caspases also influence several processes including proliferation, differentiation and cell cycle regulation (329), it can be concluded that caspases activate secondary pathways which contribute to skin blistering (154, 302). The fact that both Dsg1 and Dsg3 can be cleaved by caspase3 also would provide a mechanism how apoptotic signalling is directly interfering with desmosome turnover (Figures 3, 5).

## Rescue Pathways in Pemphigus Such as Dsg2 and cAMP Upregulation

Most interestingly, autoantibodies in pemphigus not only cause pathogenic effects leading to loss of keratinocyte adhesion but also induce rescue pathways. One compensatory mechanism is up-regulation of Dsg2 expression in PV patients' lesions (330). It was demonstrated that forced expression of Dsg2 in the upper skin layers of transgenic mice was protective against PF-IgG-induced skin lesions (331). Moreover, in cultured keratinocytes it was shown that expression of Dsg2 (221) similar to Dsg1 (332) was protective against anti-Dsg3-antibody-induced loss of adhesion. Because Dsg2 can undergo heterophilic interaction with Dsg3, which is less sensitive to direct inhibition of autoantibodies targeting Dsg3, it is possible that Dsg2 up-regulation is supporting to maintain cell adhesion in PV (333).

Another protective pathway is dependent on the second messenger cAMP. Increased levels of cAMP were observed after incubation with PV-IgG and a pharmacological increase in cAMP blocked the effects of PV-IgG *in vitro* and skin blistering in mice *in vivo* (334). It was demonstrated that cAMP was protective against activation of p38MAPK which may involve PKA activation (334). This mechanism may be related to a phenomenon referred to as positive adhesiotropy by which adrenergic signalling in the heart strengthens cardiomyocyte adhesion *via* PKA-mediated PG phosphorylation (210, 212, 214). Recently, a case of a pemphigus patient was reported who was treated with Apremilast, a phosphodiesterase-4 inhibitor which enhances intracellular cAMP levels. Treatment ameliorated the symptoms and led to a decrease in autoantibody titers (335). Thus, the protective effect was attributed to changes in immune cell signalling rather than modulation of keratinocyte signalling.

Most recently it was reported, that mechanical stimulation also plays a very important role for the resistance against pemphigus-IgG-induced effects. Stretching of cells reportedly induced activation of RohA, strengthening the cortical acting network and its crosslinking, leading to increased cell contractility. This was shown to reduce pemphigus IgG induced effects, indicating, that activation of RhoA in pemphigus might represent an insufficient rescue pathway (336) (Figures 2, 5).

## Conclusion: How to Elucidate a Feasible Approach to Stabilise Desmosomal Adhesion for Pemphigus Therapy?

From this review it can be concluded that some signalling pathways such as p38MAPK, ERK and PLC/Ca^2+^ appear to be more relevant for skin blistering in pemphigus than others. This is supported by a recent study using an unbiased approach by testing a library of 141 small molecule inhibitors in different experimental models of pemphigus which found that inhibition of ERK and p38MAPK signalling was effective to ameliorate PV-IgG-induced loss of cell adhesion (229).

On the other hand, inhibition of p38MAPK in clinical studies was not beneficial for pemphigus patients (271). This maybe explained in part by the fact that inhibition of a central signalling pathway involved in many different functions (234) is associated with severe side effects. It can be envisaged that the situation is not different for approaches modulating ERK or PLC/Ca^2+^ signalling indicating that at least systemic therapy using inhibitors of these pathways are most likely not feasible. Nevertheless, since topical application of Dsg-crosslinking peptides or drugs to increase cAMP were effective in pemphigus mouse models *in vivo* (216, 334) it may also be possible to reduce side-effects by site-specific application of modulators of signalling pathways in the future. Therefore, it is very encouraging that topical application of selumetinib to inhibit MEK1, which is the kinase up-stream of ERK1/2, was protective in the human skin organ culture model (229).

Moreover, identification of signalling mechanisms critical for pemphigus pathology allows to focus on the molecular targets of the associated signalling molecules. Because PKC downstream of PLC/Ca^2+^ has been shown to regulate desmosome stability *via* phosphorylation of DP, the modulation of DP cytoskeletal anchorage may be a promising target for therapy. Similarly, given that p38MAPK induces pathogenic effects *via* inhibition of RhoA, which *via* Rho kinase and adducin phosphorylation is important for desmosome assembly (190, 192, 193, 227), therapeutic approaches to stimulate desmosome assembly should be characterised in more detail. Finally, since the small inhibitor library helped to identify a role in keratinocyte adhesion regulation for VEGFR2, TrkA, PI3K, and PDK1 (Table 2), new candidates for treatment options have been elucidated as well (229). Therefore, we believe that studies on agents for additional treatment of patients suffering from the acute phase of pemphigus or facing relapses are of high importance in the future.

## Author Contributions

TS: concept, original draft, figure design, and revision. JW: concept and revision. Both authors contributed to the article and approved the submitted version.

## Conflict of Interest

The authors declare that the research was conducted in the absence of any commercial or financial relationships that could be construed as a potential conflict of interest.

## Publisher's Note

All claims expressed in this article are solely those of the authors and do not necessarily represent those of their affiliated organizations, or those of the publisher, the editors and the reviewers. Any product that may be evaluated in this article, or claim that may be made by its manufacturer, is not guaranteed or endorsed by the publisher.
